# Nuclear Survivin and Its Relationship to DNA Damage Repair Genes in Non-Small Cell Lung Cancer Investigated Using Tissue Array

**DOI:** 10.1371/journal.pone.0074161

**Published:** 2013-09-16

**Authors:** Songliu Hu, Yuanyuan Qu, Xiangying Xu, Qingyong Xu, Jingshu Geng, Jianyu Xu

**Affiliations:** 1 Department of Radiation Oncology, The Third Affiliated Hospital of Harbin Medical University, Harbin, China; 2 Department of Pathology, The Third Affiliated Hospital of Harbin Medical University, Harbin, China; Univesity of Texas Southwestern Medical Center at Dallas, United States of America

## Abstract

**Purpose:**

To investigate the predictive role and association of nuclear survivin and the DNA double-strand breaks repair genes in non-small cell lung cancer (NSCLC): DNA-dependent protein kinase catalytic subunit (DNA-PKcs), Ku heterodimeric regulatory complex 70-KD subunit (Ku70) and ataxia-telangiectasia mutated (ATM).

**Methods:**

The protein expression of nuclear survivin, DNA-PKcs, Ku70 and ATM were investigated using immunohistochemistry in tumors from 256 patients with surgically resected NSCLC. Furthermore, we analyzed the correlation between the expression of nuclear survivin, DNA-PKcs, Ku70 and ATM. Univariate and multivariate analyses were performed to determine the prognostic factors that inuenced the overall survival and disease-free survival of NSCLC.

**Results:**

The expression of nuclear survivin, DNA-PKcs, Ku70 and ATM was significantly higher in tumor tissues than in normal tissues. By dichotomizing the specimens as expressing low or high levels of nuclear survivin, nuclear survivin correlated significantly with the pathologic stage (P = 0.009) and lymph node status (P = 0.004). The nuclear survivin levels were an independent prognostic factor for both the overall survival and the disease-free survival in univariate and multivariate analyses. Patients with low Ku70 and DNA-PKcs expression had a greater benefit from radiotherapy than patients with high expression of Ku70 (P = 0.012) and DNA-PKcs (P = 0.02). Nuclear survivin expression positively correlated with DNA-PKcs (P<0.001) and Ku70 expression (P<0.001).

**Conclusions:**

Nuclear survivin may be a prognostic factor for overall survival in patients with resected stage I-IIIA NSCLC. DNA-PKcs and Ku70 could predict the effect of radiotherapy in patients with NSCLC. Nuclear survivin may also stimulates DNA double-strand breaks repair by its interaction with DNA-PKcs and Ku70.

## Introduction

Non-small-cell lung cancer (NSCLC) is the leading cause of cancer-related death worldwide, with an increasing incidence and a poor prognosis. As the clinico-pathologic characteristics can not accurately predict the patient outcome, understanding the biology of NSCLC at the molecular level will help in the development of new and effective treatment modalities and prediction of the prognosis.

Evasion of apoptotic cell death is critical for tumor growth and is reported to be a hallmark of cancer cells [1] that develop resistance to anticancer treatments. Thus, targeting the apoptotic pathways may represent a promising strategy to counteract resistance and sensitize the cancer cells to anticancer modalities, including radiotherapy [2]. Among these anti-apoptotic factors, survivin, a 16.5 KD protein of 142 amino acid residues that is the smallest member of the inhibitor of apoptosis protein (IAP) family, deserves much attention due to its universal over-expression in human tumors, and its prominent role in the regulation of a variety of cellular networks, including those that control tumor cell proliferation and adaption to an unfavorable environment [3]. Survivin exist in distinct subcellular pools including the nucleus, the cytoplasm and the mitochondria [4]–[6]. The subcellular distribution of survivin plays a distinct role in the ability of this molecule to regulate cell division and survival. The localization of survivin in the cytoplasm is considered to be cytoprotective due to the anti-apoptotic activities of this molecule [7]. The nuclear localization of survivin is associated with cell division, as survivin is a subunit of the chromosomal passenger complex (CPC) [8].

In mammalian cells, DNA double-strand breaks (DSBs) are mainly repaired via homologous recombination (HR) or nonhomologous end joining (NHEJ) [9]. A key element of the NHEJ pathway is the DNA-dependent protein kinase (DNA-PK), which consists of a 465-KD catalytic subunit of the DNA-dependent protein kinase (DNA-PKcs), and a heterodimeric regulatory complex, Ku,which includes a 70-KD subunit (Ku70) and an 86-KD subunit (Ku80) [10]. Current models suggest that the heterodimeric Ku proteins rapidly bind to double stranded DNA ends and recruit DNA protein kinase (DNA-PK), generating a DNA-PK holoenzyme complex. The ATM protein has been identified as the principal activator and master controller of the cellular response to DSBs. The ATM kinase seems to be the primary activator and master controller of the cellular response to DNA DSBs and phosphorylates key players of the DNA damage response network, including cell cycle arrest apoptosis and DNA repair [11].

Recently it was shown that survivin is able to provoke both suppression of apoptosis and stimulation of DNA repair. The underlying mechanisms seem to be multifaceted and involve caspase-dependent and caspase-independent pathways. Some reports confirmed a higher incidence of DNA-damage in colorectal- and non-small cell lung cancer cell lines after treatment with survivin siRNA or the transcriptional repressor YM155 based on the detection of phospho-histone γ-H2AX detection as a marker of radiation-induced DSBs [12], [13]. Co-immunoprecipitation analyses revealed an interaction between survivin, Ku70 and DNA-PKcs in nuclear foci [14]. These results suggested that the nuclear accumulation of survivin was linked to DNA-DSB repair.

The aim of this study was to examine the potential correlation and prognostic value of nuclear survivin, DNA-PKcs, Ku70 and ATM using immunohistochemistry. To our knowledge, this is the first study.

## Materials and Methods

### Patients

The study was approved by the Ethical Review Committee of Harbin Medical University, Harbin, China. All patients provided written informed consent to participate in the study. This study was retrospective. Surgical resection specimens were obtained from 256 lung cancer patients at stage IA to IIIB between December 2004 and December 2006. No patients received pre-operative chemotherapy and radiotherapy. There were 176 males and 80 females with a mean age of 57.7 yrs. The tumors were staged according to the revised version of lung cancer TNM staging published in 1997 [15]. Histopathological diagnosis was performed according to the World Health Organization (WHO) criteria. In total, 20 (8%) patients had stage IA disease, 94 (35%) had IB, 8 (3%) had IIA, 50 (20%) had IIB, 69 (27%) had IIIA, and 15 (6%) had IIIB. The histologic classification included 145 adenocarcinomas, 101 squamous cell carcinomas, 5 adenosquamous carcinomas and 5 other types. In total, 234 patients underwent lobectomy, 18 patients underwent pneumonectomy and 4 patients underwent segmentectomy. After surgery, 217 patients received three to four cycles of adjuvant cisplatin-based chemotherapy. A total of 92 patients received postoperative radiotherapy administered with a high-voltage technique at a total dose of 50 Gy, with 2 Gy per fraction given 5 days per week.

### Construction of Tissue Microarrays

The lung cancer tissue microarray (TMAs) was constructed as follows. Briefly, a tissue arraying instrument was used to create holes in a receptive paraffin block and to acquire tissue cores from the donor tissue block using a thin-walled needle with an inner diameter of 2 mm that was held in an X-Y precision guide. The core samples were retrieved from the selected region in the donor and extruded directly into the receptive block at defined array coordinates. A solid steel wire that was closely fit in the tube, was used to transfer the tissue cores into the receptive block. After the construction of the array block, all the tissue blocks were cut with a microtome to 4 µm and affixed to the slide. Blocks from 256 patients were arrayed as triplicate spots of 2 mm diameter on slides.

### Immunohistochemistry

The tissue sections were deparaffinized in xylene and rehydrated with graded alcohol concentrations using standard procedures. The sections were subsequently submerged in citrate buffer (pH 6.0) and autoclaved at 121°C for 5 min to retrieve the antigenicity. After washing in phosphate-buffered saline (PBS,0.1 M,PH 7.4,3 times for 5 min), endogenous peroxidase was blocked by incubation in 3% hydrogen peroxide for 15 min at room temperature. Then, the samples were incubated with a survivin antibody (71G4B7,Cell Signaling Technology, Beverly, MA) diluted at 1∶400, a DNA-PKcs antibody (MS-423-P0,Neomarkers, Fremont, CA), a Ku70 antibody (EPR4027,Epitomics, CA) and an ATM antibody (Y170,Epitomics, CA) diluted at 1∶200 in PBS containing 0.5% BSA overnight at 4°C in a moist chamber and washed with buffer to remove unbound antibodies. The sections were incubated with the biotinylated secondary antibody followed by peroxidase-conjugated streptavidin for 30 min. 3-3′diaminobenzidine tetrahydrochloride (Dako,Germany) was added to visualize the reaction. After rinsing in deionized water and counterstaining with commercial hematoxylin, the slides were dehydrated and mounted. Appropriate tissue sections as positive controls for each primary antibody were simultaneously stained to be used as positive controls. Mouse IgG1 or rabbit IgG (Cell Signaling Technology, Beverly, MA) were used as negative controls at the same dilutions as the corresponding primary antibodies.

### Immunohistochemical Staining Evaluation

Evaluation of the staining for survivin, DNA-PKcs, Ku70 and ATM expression was conducted with bright-field light microscopy independently by two experienced pathologists independently. Nuclear survivin, DNA-PKcs,Ku70 and ATM protein expression levels were classified semiquantitatively by combining the proportion and intensity of the positively stained tumor cells. The percentage of positively stained tumor cells was scored as follows: 0 (no positive tumor cells), 1 (1–25% positive tumor cells), 2 (26–50% positive tumor cells), 3 (51–75% positive tumor cells), and 4 (76–100% positive tumor cells). Staining intensity was scored as follows: 0(no staining); 1(weak staining); 2(moderate staining) and 3(strong staining). The staining intensity score multiplied by the percentage of positive staining was used to define the expression levels. The mean score among the three tissue sections was entered for statistical analyses. Subsequently, the median value of all scores was used as the cut-off point for the classification of the expression of the 4 proteins, thus lung cancer patients were classified into two groups: low expression and high expression groups. Furthermore, cases with discrepancies were reviewed simultaneously by the original two pathologists and a senior pathologist until a consensus was reached.

### Statistical Analysis

The associations between the IHC parameters, as well as the IHC/clinical-pathological parameters were analyzed using the Chi-squared test, the Fisher exact test and the Pearson rank correlation. Overall survival (OS), disease-free survival (DFS) and local recurrence-free survival (LRS) were obtained according to the Kaplan-Meier method. The persistence of the disease and the occurrence of relapses were the end points for DFS, the occurrence of local recurrence was the end point for LRS, and death from any cause was considered an event for the calculation of OS. The comparisons between each prognostic variable were conducted using the Log-rank test. A Cox proportional hazards model was used for multivariate analyses of the independent prognostic factors (age, sex, smoking status, histology, differentiation grade, pathologic stage, T stage, N stage, chemotherapy, radiotherapy, survivin expression, DNA-PKcs expression, Ku70 expression and ATM expression) influencing the OS, DFS. The criterion of significance chosen was p<0.05, and all tests were two-tailed. The survival analysis was conducted using the statistical package for SPSS version 13.0.

## Results

**Figure 1:**
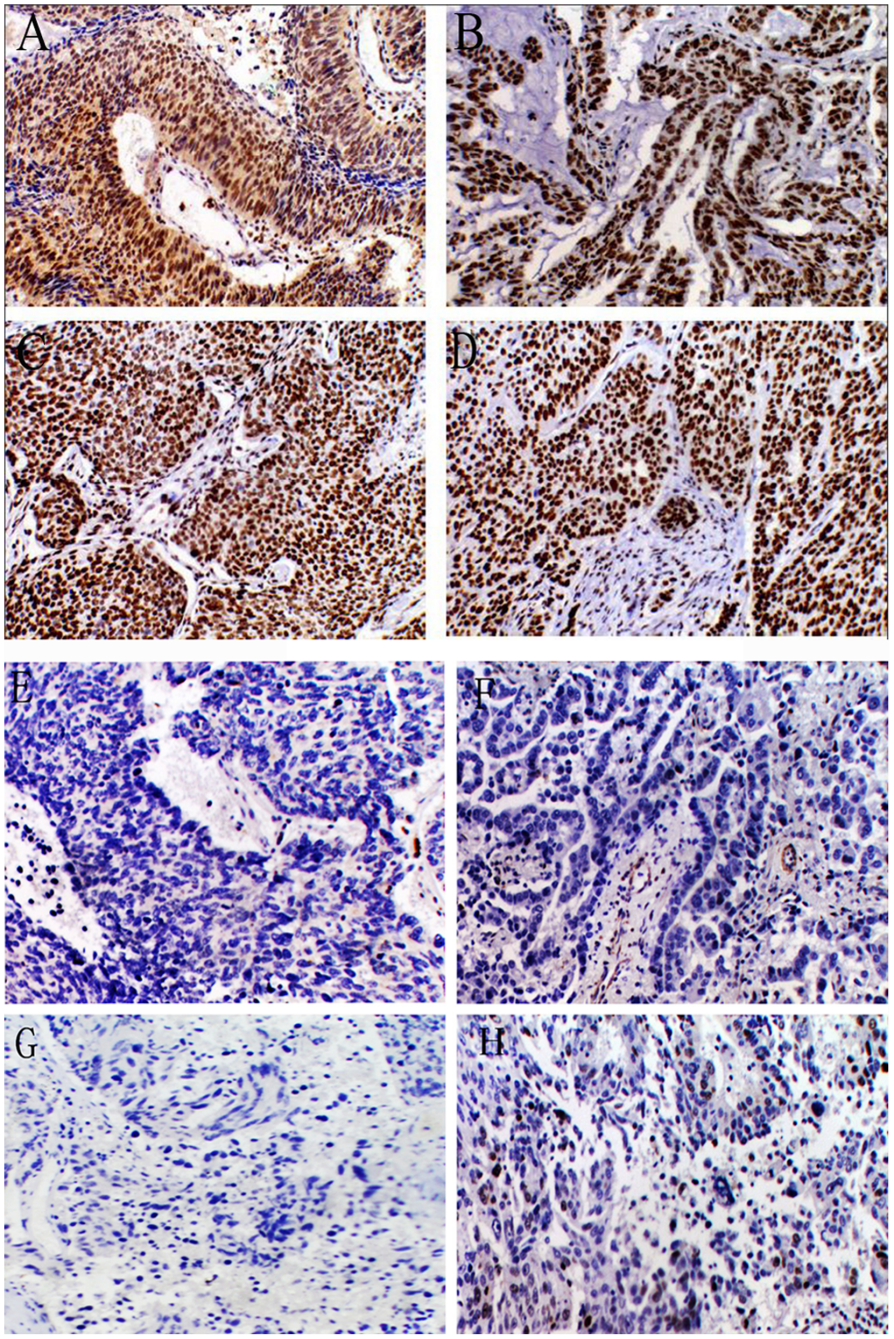
Strong staining for ATM (A), DNA-PKcs (B), Ku70 (C) and nuclear survivin (D) in lung cancer tissues. Weak staining for ATM (E), DNA-PKcs (F), Ku70 (G) and nuclear survivin (H) in lung cancer tissues.

Nuclear survivin immunoreactivity was detected in 166(64.8%) out of the 256 tumors examined. Positive immunoreactivity for survivin was present only in tumor cells and not in the neighboring normal lung epithelial cells. In tumor tissues, the staining for DNA-PKcs, Ku70 and ATM was predominantly nuclear with minute staining in the cytoplasm. The normal tissues showed a weak staining intensity for ATM and a moderate staining intensity for DNA-PKcs and Ku70. The percentage of positive nuclei was significantly higher in the tumor tissue compared with the normal tissue. DNA-PKcs immunoreactivity was observed in 190 (74.2%) out of 256 tumors and 56 (51.9%) out of 108 normal tissues; Ku70 immunoreactivity was observed in 189 (73.8%) out of 256 tumors and 48 (44.4%) out of 108 normal tissues, and ATM immunoreactivity was observed in 125 (48.8%) out of 256 tumors and 26 (24.1%) out of 108 normal tissues. When the tumors were categorized according to the staining intensity score and the positive staining score, 124 tumors had low expression and 132 had high expression of survivin, 121 tumors had low expression and 135 had high expression of DNA-PKcs, 107 tumors had low expression and 149 had high expression of Ku70 and 156 tumors had low expression and 100 had high expression of ATM (Fig. 1).

### Correlation with Prognostic Factors

The correlations between patients expressing nuclear survivin, DNA-PKcs, Ku70 and ATM at different levels and their clinico-pathological characteristics were presented in Table 1. Nuclear survivin correlated significantly with the pathologic stage and lymph node status. Nuclear survivin expression levels in patients with stage III tumors were markedly higher than those in patients with stage I and stage II tumors (P = 0.009). Nuclear survivin expression in patients with N2 and N3 disease was higher than those in patients with N0 and N1 disease (P = 0.004). There was no statistically significant difference between DNA-PKcs, Ku70 or ATM expression and the clinicopathologic variables.

**Table 1:** Patient clinico-pathologic characteristics in relation to gene expression.

		Survivin			DNA-PKcs			Ku70			ATM		
		Low	High	P	Low	High	P	Low	High	P	Low	High	P
**Age**													
≤57	127	63	64	0.71	62	65	0.621	51	76	0.598	81	46	0.355
>57	129	61	68		59	70		56	73		75	54	
**Sex**													
male	176	83	93	0.544	81	95	0.555	77	99	0.347	104	72	0.369
female	80	41	39		40	40		30	50		52	28	
**Histology**													
squamous cell carcinoma	101	52	49		51	50		50	51		59	42	
adenocarcinoma	145	69	76	0.536	68	77	0.332	55	90	0.073	93	52	0.220
adenosquamous carcinoma	5	1	4		1	4		2	3		3	2	
other	5	2	3		1	4		0	5		1	4	
**Differentiation grade**													
poor	124	60	62		61	61		50	72		69	53	
moderate/well	115	56	59	0.846	52	63	0.683	50	65	0.836	76	39	0.162
undefined	19	8	11		8	11		7	12		9	10	
**Pathologic stage**													
I	114	66	48		59	55		54	60		68	46	
II	58	28	30	0.009	28	30	0.287	24	34	0.193	37	21	0.869
III	84	30	54		34	50		29	55		51	33	
**T stage**													
T1	35	19	16		14	21		20	15		24	11	
T2	184	92	92	0.105	88	96	0.349	75	109	0.160	113	71	0.455
T3	32	13	19		18	14		11	21		17	15	
T4	5	0	5		1	4		1	4		2	3	
**N stage**													
N0	134	79	55		65	69		66	68		75	59	
N1	47	20	27	0.004	24	23	0.795	15	32	0.057	33	14	0.201
N2	65	22	43		28	37		24	41		40	25	
N3	10	3	7		4	6		2	8		8	2	

### Expression of Nuclear Survivin and DNA DSB Repair Genes in Relation to Prognosis

The median follow-up time was 64 months. The median OS of the patients was 47±5.8 months, and the median DFS time was 25±4.5 months. Univariate survival analysis revealed that the tumor histology, differentiation grade, pathologic stage, T status, N status, nuclear survivin expression, DNA-PKcs expression and Ku70 expression significantly influenced the OS and DFS (Table 2). Multivariate analysis was performed according to the results of the univariate analysis. The results indicated that the tumor differentiation grade, pathologic stage and nuclear survivin expression were the independent prognostic factors (Table 3). The calculated relative risks of death and recurrence was 1.68 (P = 0.047) and 1.83 (P = 0.041) for patients with high survivin expression, 1.67 (P = 0.001) and 1.59 (P = 0.003) for patients with a high pathologic stage and 0.63 (P = 0.015) and 0.51 (P = 0.002) for patients with low differentiation, respectively.

**Table 2:** Univariate analysis with prognostic factors.

Variable	No	OS			DFS		
		Median	95% CI	P	Median	95% CI	P
**Age**							
≤57	127	49.9	44.6–55.2	0.941	42.0	36.2–47.8	0.945
>57	129	47.8	42.6–53.0		42.6	36.8–48.4	
**Sex**							
male	176	48.7	44.2–53.3	0.639	41.6	36.6–46.5	0.601
female	80	50.9	44.1–57.6		41.4	34.7–48.1	
**Smoking status**							
never	111	54.2	46.1–62.4	0.083	44.3	35.9–52.7	0.065
ever	145	43.7	36.7–50.6		35.6	28.0–43.3	
**Histology**							
adenocarcinoma	145	44.9	39.9–49.8		36.0	30.9–41.1	
squamous cell carcinoma	101	57.0	51.1–62.8	0.019	51.1	44.7–57.5	0.011
adenosquamous carcinoma	5	36.6	16.6–56.6		22.2	10.7–33.7	
other	5	34.4	11.0–57.8		31.4	5.8–57.0	
**Differentiation grade**							
moderate/well	122	54.5	49.0–60.0		48.0	42.1–54.1	
poor	115	43.1	37.6–48.5	0.007	35.3	29.6–41.1	0.008
undefined	19	51.2	39.0–63.3		41.5	29.1–54.0	
**Pathologic stage**							
I	114	61.4	56.1–66.6		55.8	50.0–61.5	
II	58	46.2	39.0–53.4	<0.001	39.3	31.0–47.5	<0.001
III	84	34.1	28.3–40.0		24.1	18.7–29.5	
**T stage**							
T1	35	59.3	50.9–67.6		54.6	44.6–64.5	
T2	184	50.5	46.1–55.0	<0.001	43.1	38.2–48.0	<0.001
T3	32	30.0	22.8–37.2		24.7	17.1–32.3	
T4	5	19.8	8.6–31.0		14.0	5.8–22.2	
**N stage**							
N0	134	57.7	52.6–62.7		52.0	46.6–57.5	
N1	47	47.7	39.9–55.4	<0.001	40.1	31.0–49.1	<0.001
N2	65	34.2	27.7–40.6		24.3	18.0–30.6	
N3	10	30.8	13.7–47.9		21.0	7.8–34.2	
**chemotherapy**							
yes	217	51.6	47.6–55.7	0.004	44.4	40.0–48.8	0.005
no	39	35.6	30.0–44.2		27.1	19.0–35.1	
**radiotherapy**							
yes	92	46.2	40.0–52.4	0.222	44.5	39.3–49.7	0.207
no	164	50.0	45.5–54.6		38.3	31.7–45.0	
**Survivin**							
low	124	55.7	50.2–61.2	0.001	49.7	43.8–55.6	0.001
high	132	42.8	38.0–47.7		35.7	30.3–41.1	
**DNA-PKcs**							
low	121	54.7	49.3–60.1	0.01	48.2	42.3–54.0	0.01
high	135	45.0	39.7–50.2		32.7	28.2–37.3	
**Ku70**							
low	107	55.7	50.4–61.0	0.002	50.4	44.3–56.5	0.002
high	149	44.4	39.4–49.3		34.1	29.3–38.9	
**ATM**							
low	156	52.0	47.3–56.7	0.096	45.2	40.0–50.3	0.086
high	100	45.5	39.3–51.7		37.8	31.3–44.4	

Abbreviations: DFS = disease-free survival; OS = overall survival.

**Table 3:** Multivariate analysis with independent prognostic factors.

	OS			DFS		
	RR	95% CI	P	RR	95% CI	P
**Differentiation** **grade**	0.63	0.44–0.92	0.015	0.51	0.33–0.79	0.002
**Pathologic stage**	1.67	1.25–2.23	0.001	1.59	1.17–2.18	0.003
**Nuclear survivin**	1.68	1.01–2.81	0.047	1.83	1.03–3.26	0.041

Abbreviations: RR = relative risk; CI = confidence interval.

**Figure 2:**
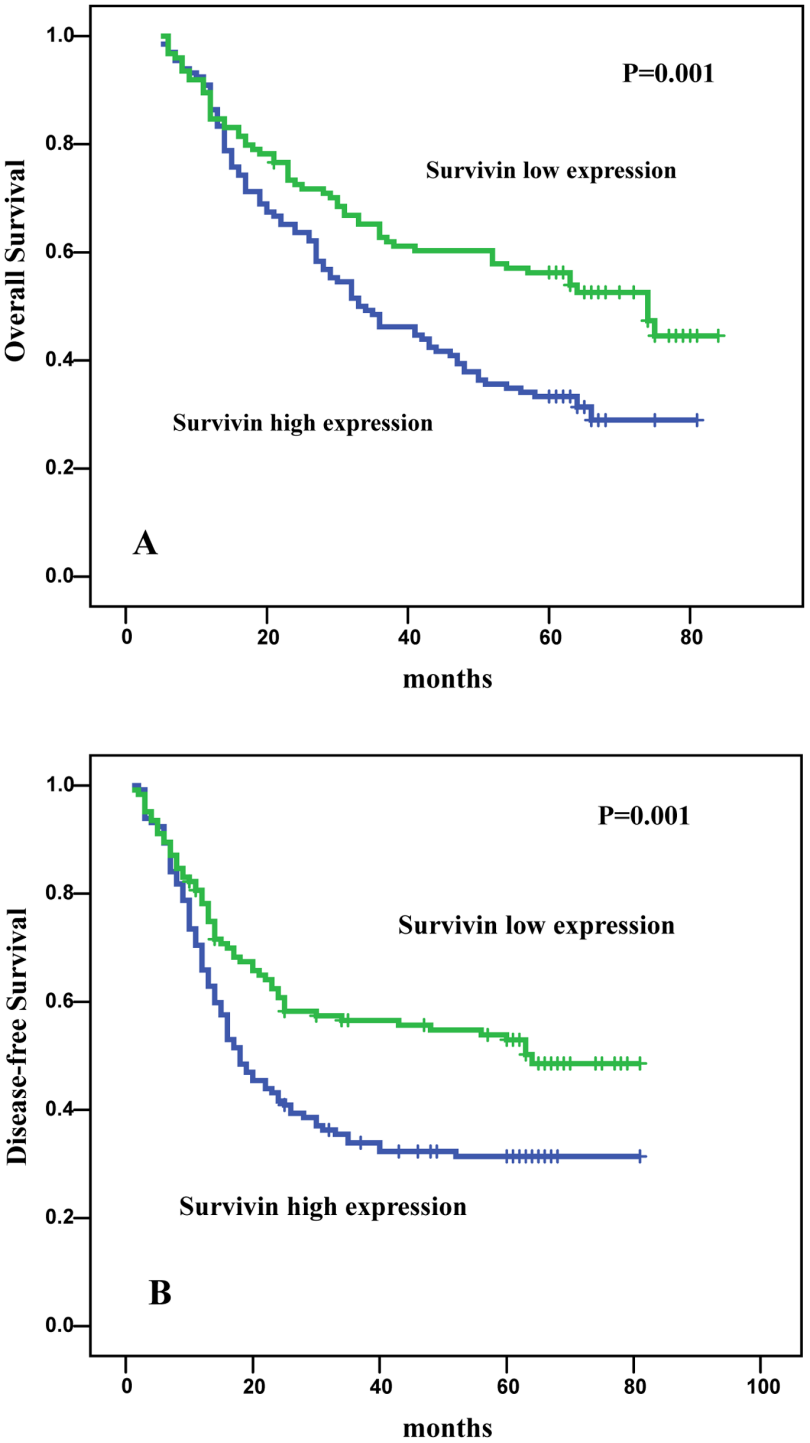
Kaplan-Meier survival curves for patients with non-small cell lung cancer according to nuclear survivin expression. A. Overall survival of patients with low nuclear survivin (n = 124)compared with that of patients with high nuclear survivin (n = 132). The P-value for the Log-Rank test is 0.001.B. Disease-free survival of patients with low nuclear survivin (n = 124)compared with that of patients with high nuclear survivin (n = 132). The P-value for the Log-Rank test is 0.001.

Kaplan-Meier analysis revealed that patients with nuclear survivin high expression had poorer OS (44.8 vs. 55.7 months; P = 0.001) (Fig. 2A) and DFS (35.7 vs. 49.7 months; P = 0.001) (Fig. 2B) compared with patients with lower levels of nuclear survivin. Patients with a moderate or strong differentiation grade had a better survival than patients with low differentiation grade (OS, P = 0.007; DFS, P = 0.008). Moreover, higher pathologic stage (OS, P<0.001; DFS, P<0.001) were significantly associated with poor OS and DFS. Patients with high DNA-PKcs or Ku70 expression had a significantly worse OS and DFS than those with lower expression but they were not independent prognostic factors.

### Relationship between Nuclear Survivin and DNA Damage Repair Genes

We investigated the possible relationship between nuclear survivin and DNA damage repair genes among the 256 patients. Among the three relevant genes, DNA-PKcs and Ku70 expression were significantly positively related to nuclear survivin expression (Table 4). In total, 91 (67.4%) out of 135 patients with high DNA-PKcs who had concurrent high survivin expression compared with 44 (32.6%) out of 135 patients with high DNA-PKcs who had low survivin expression (P<0.001). In total, 99 (66.4%) out of 149 patients with high Ku70 had concurrent high survivin expression compared with 50 (33.6%) out of 149 patients with high Ku70 expression and low survivin expression (P<0.001). In survivin negative cases, DNA-PKcs expression significantly influenced the OS (46.0 vs. 61.3 months; P = 0.009) and DFS (35.0 vs. 55.1 months; P = 0.02). This was not found in survivin positive cases.

**Table 4:** Correlation between the expression of nuclear survivin and DNA repair proteins.

	DNA-PK		KU70		ATM	
Nuclear survivin	Low	High	Low	High	Low	High
low	80	44	74	50	77	47
high	41	91	33	99	79	53
P	0.000		0.000		0.714	
CC	0.709		0.528		0.023	

Abbreviations: CC: correlation coefficient.

### Prediction of Treatment Outcome

**Figure 3:**
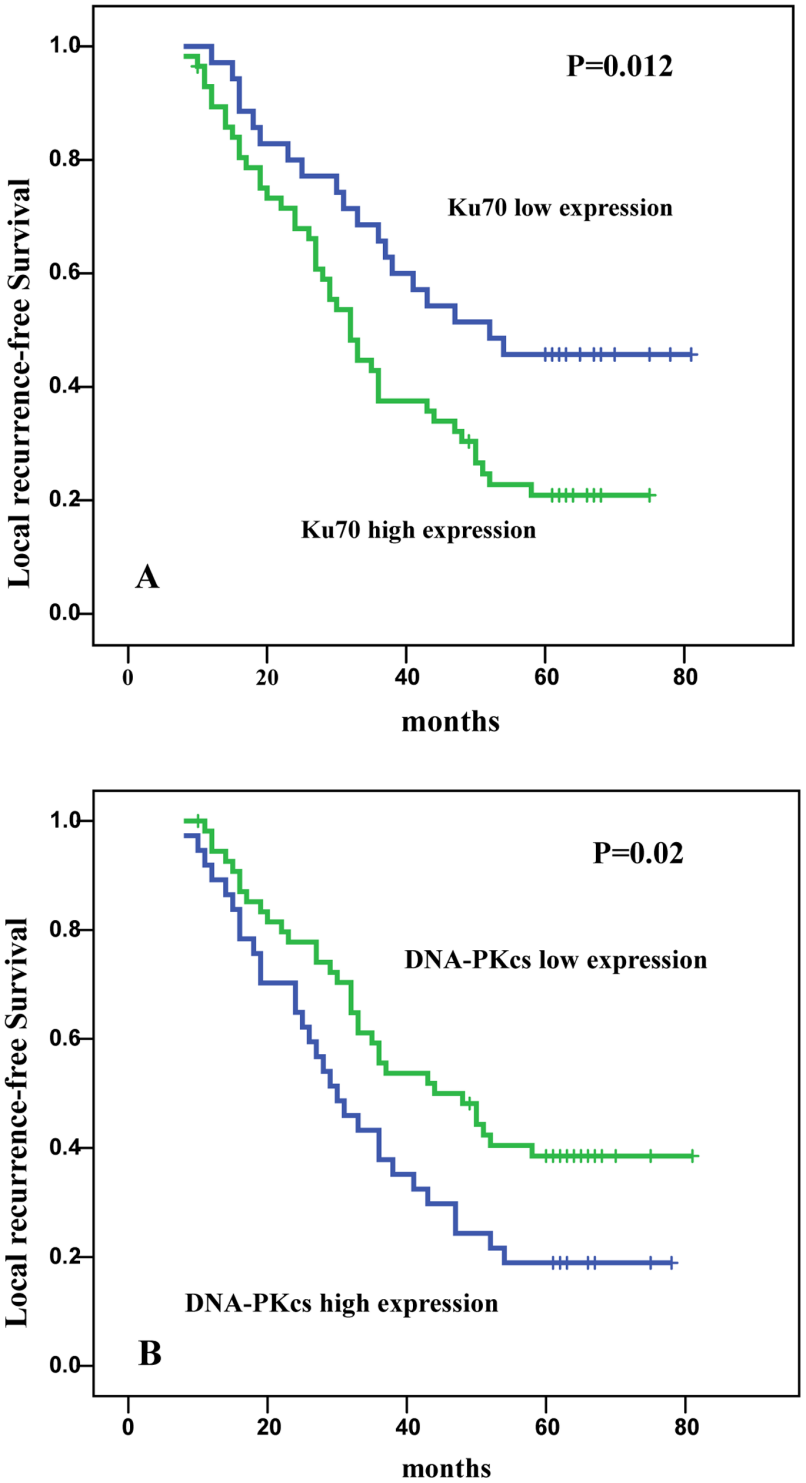
Local recurrence-free survival for patients with non-small cell lung cancer according to Ku70 expression and DNA-PKcs expression. A. Local recurrence-free survival of patients with low Ku70 expression (n = 36)compared with that of patients with high Ku70 survivin (n = 56). The P-value for the Log-Rank test is 0.012. B. Local recurrence-free survival of patients with low DNA-PKcs expression (n = 49)compared with that of patients with high nuclear survivin (n = 53). The P-value for the Log-Rank test is 0.02.

Postoperative radiotherapy could significantly reduce the risk of local recurrence. In our study, 92 patients received postoperative radiotherapy. Multivariate analysis indicated that the tumor differentiation grade, DNA-PKcs and Ku70 expression were the independent prognostic factors (Table 5). The calculated relative risks of local recurrence was 2.025(P = 0.007) for patients with high DNA-PKcs expression, 2.161(P = 0.029) for patients with high Ku70 expression and 0.522 (P = 0.008) for patients with low differentiation grade, respectively. In patients with low expression of Ku70 and DNA-PKcs, the local recurrence-free survival (LRS) was significantly higher compared to patients with high expression of Ku70 (P = 0.012; Figure. 3A) and DNA-PKcs (P = 0.02; Figure. 3B). The greatest benefit of radiotherapy was observed in patients whose tumors had low expression of Ku70 and DNA-PKcs.

**Table 5:** Relative risk of local recurrence in the radiotherapy-treated group.

	B	SE	Wald	RR	95% CI	P
**Differentiation grade**	0.65	0.245	7.022	0.522	0.323–0.844	0.008
**DNA-PKcs**	0.706	0.263	7.214	2.025	1.120–3.389	0.007
**Ku70**	0.770	0.353	4.776	2.161	1.083–4.312	0.029

Abbreviations: RR = relative risk; CI = confidence interval.

## Discussion

In the current study, we determined the expression of nuclear survivin and 3 key components of the DNA DSB repair pathways in patients with NSCLC using TMAs. Compared with normal lung tissue, the proportion of cells with positively stained nuclei and the staining intensity were higher in tumor tissues than in normal lung tissue. Reduced expression of messenger RNA for DNA-PKcs and ATM in normal lung tissue has also been shown in a previous study [16]. Survivin expression was only observed in tumor samples, not in the surrounding normal tissues, which is in accordance with the results of previous studies [17], [18].

The prognostic predictive value of survivin expression and that of nuclear staining versus cytoplasmic staining in several malignancies, including NSCLC, have been investigated in a number of studies with conflicting results. Similarly, the results of studies investigating the prognostic predictive value of the nuclear staining of survivin are also conflicting. These controversies could be explained, in IHC studies, on the basis of using antibodies of different specificities or concentrations, and employing different cut-off points and different approaches for storing and processing tissues. Our study showed that the nuclear expression of survivin is an independent negative prognostic factor for survival in surgically resected NSCLC patients. Consistent with our results, other retrospective series have identified nuclear rather than cytoplasmic survivin expression as an independent negative prognostic factor for survival in NSCLC [19]–[22]. Even in NSCLC, nuclear survivin expression was shown to be correlated with a better outcome in a recent series of advanced (stage IIIA–IV) inoperable patients who underwent chemotherapy [17]. However, a meta-analysis of survivin conducted by Jiang Fan [23] using individual patient data found conflicting results. Data from three studies were combined to find that the level of nuclear survivin did not have impact on OS of NSCLC patients (RR1.58, 95% CI 0.87–2.85, P = 0.13). But the studies only included three studies and the number of the patients were small, the negative result may be due to the small number of patients.

In our study, nuclear survivin expression positively correlated with the pathologic stage and the lymph node status. The current results are consistent with those that reported that survivin was a marker of lymph node metastasis [18], [24], [25]. The link between survivin expression and the potential for lymph node metastasis could be explained as follows. Survivin could be an apoptosis inhibitor: the proportion of cancerous cells in a tissue, which would otherwise be removed by apoptosis, increase with continued growth and increased potential for invasion and metastasis.

In this study, we observed that high expression of DNA-PKcs and Ku70 was associated with poor overall survival in patients with NSCLC but it did not reach statistical significance. DNA double-strand breaks (DSBs) are regarded as the most lethal of all DNA lesions. Prompt and efficient repair of DNA DSBs is critical for maintaining genomic integrity. Defects in the DSB repair pathway may cause genetic alterations, chromosome instability, and, ultimately, malignant transformation. Conversely, tumor cells with increased DNA DSB repair capacity would be more likely to survive and proliferate, leading to the poor prognosis in cancer patients. The prognostic value of these genes were investigated and the results were conflicting. In the study conducted by Jinliang et al in which RT-PCR was used to measure DNA-PKcs and ATM expression in lung cancer, patients with high tumor:normal (T/N) expression ratios of ATM or DNA-PKcs had a notably shorter median survival than patients with low ratios [16]. However, there are also some inconsistent results in terms of the prognostic value of these genes. In a comprehensive review, Wynand reported that cells that exhibit a defect in their DNA repair pathways are liable to suffer from DNA damage-induced apoptosis [26]. For example, the survival of patients with high expression of DNA-PKcs or Ku86 was significantly better when compared with the survival of patients with low expression of DNA-PKcs or Ku86 [27]. These inconsistencies may be the result of many factors, including different cancer types, different histology and tumor characteristics, or methodological differences, such as the technique used to probe gene expression or the antibody reacting against a specific epitope.

The molecular mechanism underlying the differential localization in tumor cells is still unclear. The subcellular distribution of survivin plays a distinct role in the ability of the molecule to regulate cell division. Recent data further indicate that the interaction of the leucine-rich nuclear export signal (NES) with the nuclear export receptor chromosome region maintenance protein 1 homolog (Crm1) is critically involved in survivin’s intracellular localization and cancer-related functions [28], [29]. DNA damage can stimulate a rapid discharge of the mitochondrial pool of survivin into the cytosol, which preserves the viability of tumor cells during a protracted G2 block by antagonizing DNA damage-induced apoptosis [30]. Using Pearson correlation coefficient analysis, the correlation between the expression levels of nuclear survivin and those of DNA-PKcs or Ku70 was observed in our study. Survivin is a member of the inhibitor of apoptosis (IAP) family, and it directly inhibits the activity of caspases, notably caspase-3. Recently, it was shown that survivin also stimulates DSB repair by its interaction with DNA-PK [14], [31]. Therefore, we speculate that DNA damage stimulates a rapid discharge of survivin to the nucleus, which physically interacts with factors of the DNA DSB repair proteins DNA-PKcs and Ku70. Thus survivin stimulates DNA double-strand repair capacities by regulating DNA-PKcs and Ku70 activity and, thereby inhibit DNA damage-induced apoptosis. However, more studies are required to clarify this open question.

The novel finding in our study is that the protein expression levels of DSB repair proteins can be of value for the prediction of treatment outcome. Patients with low Ku70 and DNA-PKcs expression had a greater benefit from radiotherapy than patients with high expression of Ku70 and DNA-PKcs because the latter group had a high rate of recurrence after treatment with radiotherapy. Our interpretation is that Ku70 and DNA-PKcs are necessary for DNA damage recognition and signaling and that loss of these proteins would lead to impaired DNA repair, increased radiosensitivity, and improved local tumor control. Karin et al. reported that low expression of Ku70/Ku80 predicted a good effect of radiotherapy in early breast cancer [32]. Beskow also found that radioresistant cervical cancer showed an increased frequency of DNA-PKcs, Ku70 and Ku86 positive cells [33]. Bouchaert et al. reported that positive DNA-PKcs nuclear staining was closely associated with biochemical recurrence and suggested that DNA-PKcs could be a predictive marker of recurrence after radiotherapy in prostate cancer [34]. However, there are also results that are inconsistent with our findings [27], [32], [35]. It remains controversial whether DNA-PKcs or Ku70 expression could be a prognostic factor for the response of patients with lung cancer to radiotherapy.

In conclusion, we observed that nuclear survivin was significantly associated with poor survival in patients with NSCLC and that nuclear survivin was strongly related to pathologic stage and lymph node metastasis. Nuclear survivin was positively correlated with DNA-PKcs and Ku70 and thus may stimulate DNA double-strand breaks repair by its interaction with DNA-PKcs and Ku70. The current results indicate that nuclear survivin may be useful for predicting the clinical outcomes of patients with NSCLC.
