# Correction: Nuclear Survivin and Its Relationship to DNA Damage Repair Genes in Non-Small Cell Lung Cancer Investigated Using Tissue Array

**DOI:** 10.1371/annotation/03b700e2-348b-4b1c-b068-4760c32de19e

**Published:** 2013-10-11

**Authors:** Songliu Hu, Yuanyuan Qu, Xiangying Xu, Qingyong Xu, Jingshu Geng, Jianyu Xu

The correct corresponding author for the paper should be Xiangying Xu. The email address given is correct. 

