# Correction: Nuclear Survivin and Its Relationship to DNA Damage Repair Genes in Non-Small Cell Lung Cancer Investigated Using Tissue Array

**DOI:** 10.1371/annotation/a87d46d5-04ee-4c57-af2b-7b9af5166cd8

**Published:** 2013-12-20

**Authors:** Songliu Hu, Yuanyuan Qu, Xiangying Xu, Qingyong Xu, Jingshu Geng, Jianyu Xu

An affiliation for the third author is missing. In addition to institution number 1, Xiangying Xu is also affiliated with the following institution: Institute of Cancer Prevention and Treatment, Harbin Medical University, Harbin, China. 

